# Sexual dimorphism in digital dermatoglyphic traits among Sinhalese people in Sri Lanka

**DOI:** 10.1186/1880-6805-32-27

**Published:** 2013-12-30

**Authors:** Buddhika TB Wijerathne, Geetha K Rathnayake, Shamila C Adikari, Subashini Amarasinghe, Prasanna L Abhayarathna, Ajith S Jayasena

**Affiliations:** 1 Department of Forensic Medicine, Faculty of Medicine and Allied sciences, Rajarata University of Sri Lanka, Saliyapura, Anuradhapura, Sri Lanka; 2 Teaching Hospital Anuradhapura, Anuradhapura, Sri Lanka; 3 Rehabilitation Hospital Digana, Digana, Sri Lanka; 4 Teaching Hospital Kandy, Kandy, Sri Lanka; 5 General Hospital Trincomale, Trincomale, Sri Lanka; 6 General Hospital Matale, Matale, Sri Lanka

**Keywords:** Digital dermatoglyphic traits, Dermatoglyphic pattern indices, Sexual dimorphism, Anthropology, Sinhalese, Sri Lanka

## Abstract

**Background:**

The purpose of this study was to evaluate gender-wise diversity of digital dermatoglyphic traits in a sample of Sinhalese people in Sri Lanka.

**Findings:**

Four thousand and thirty-four digital prints of 434 Sinhalese individuals (217 males and 217 females) were examined for their digital dermatoglyphic pattern distribution. The mean age for the entire group was 23.66 years (standard deviation = 4.93 years). The loop pattern is observed more frequently (n = 2,592, 59.72%) compared to whorl (n = 1,542, 35.53%) and arch (n = 206, 4.75%) in the Sinhalese population. Females (n = 1,274, 58.71%) have a more ulnar loop pattern than males (n = 1,231, 56.73%). The plain whorl pattern is observed more frequently in males (n = 560, 25.81%) compared to females (n = 514, 23.69%).The double loop pattern is observed more frequently on the right and left thumb (digit 1) of both males and females. Pattern intensity index, Dankmeijer index and Furuhata index are higher in males.

**Conclusions:**

Ulnar loop is the most frequently occurring digital dermatoglyphic pattern among the Sinhalese. All pattern indices are higher in males. To some extent, dermatoglyphic patterns of Sinhalese are similar to North Indians and other Caucasoid populations. Further studies with larger sample sizes are recommended to confirm our findings.

## Background

Dermatoglyphics (ancient Greek, derma = skin, glyph = craving) [1] is the term applied to the scientific study of fingerprints. Fingerprints are characterized by alternating strips of raised friction ridges and grooves. In 1926, Harold Cummins introduced the term‘dermatoglyphic’ and he is considered to be the father of American fingerprint analysis [2] although Sir Francis Galton had linked dermatoglyphics with genetics in 1892 [3]. These patterns start to develop between the fifth and sixth week of intrauterine life, and are fully formed by the 21st week [4]. These patterns do not change throughout postnatal life and remain unique to any individual [3]. Hence, it has been used widely in the fields of forensic medicine, medicine, anthropology, ethnology and genetics. In 1961, Cummins and Midlo, after their dermatoglyphic study on various racial samples,pointed out that dermatoglyphic characters of females differ from males universally, although sexual distinction may be leveled or even inverted in some populations [2,5]. They observed a higher frequency of ulnar loops and arches buta lower frequency of whorls and radial loops in females compared to males.

To date, sexual dimorphism of qualitative dermatoglyphic traits has been studied in various populations around the world. In 1892, Sir Francis Galton examined 5,000 digital prints from different populations in which he observed the pattern distribution as loop (67.5%), whorl (26%) and arch (6.55%) patterns [3]. Chattopadhyay *et al*. [6], in their study on Rarhi Brahmins in Bengal, found that loop pattern was the most common pattern followed by whorl and arch in both males and females. However Biswas [7], in his study, found that whorl pattern was the most common pattern among Dhimals of North Bengal followed by loop and arch and Banik *et al*.’s [8] study on Rengma Nagas of Nagaland in India, observed that whorl pattern was most common followed by loop and arch in both gender. Nithin *et al*. [9], in their study on South Indian people, observed that loop pattern was more frequent than whorl and arch and Srivastava [10], in his study on Danguria Tharu of Uttar Pradesh in India,also found that loop pattern was the most common pattern followed by whorl and arch. Tiwari *et al.*’s [11] study on Tibetans in Tibet, found that whorl was the most common pattern followed by loop and arch in males, whereas in females, loop pattern was the most common pattern followed by whorl and arch and Cho’s [12] study on Samoan New Zealanders in New Zealand observed that whorl was the most common pattern followed by loop and arch. Another study done by Cho [13] among the Aborigines of the Northern Territory in Australia, also found that whorl was the most common pattern followed by loop and arch, which contrasted with Igbigbi *et al.*’s [14] study on Indigenous black Zimbabweans, which observed that loop pattern was the most common pattern followed by whorl and arch. Similarly, the study on Muzziena Bedouin in South Sinai by Karmakar *et al*. [15] observed that loop was the most common pattern in males followed by whorls and arch, whereas whorl pattern was found more frequently in females followed by loops and arch. Namouchi [16], in her study on Tunisians of Tunisia observed that loop was the most common pattern followed by whorl and arch in both males and females, which was similar to the study by Qazi *et al*. [17] whose study on Black Americans in USA, found that loop was the most common pattern followed by whorl and arch in both sexes. Finally, Boroffice [18], in his study on Nigerians observed that loop was the most common pattern followed by whorl and arch in both genders.

Dermatoglyphic data of Sinhalese people (an Indo-Aryan ethnic group native to the island of Sri Lanka) are scarce in the literature. The main objectives of the current study are to determine the sexual dimorphism of digital dermatoglyphic traits and pattern indices in a sample of the Sinhalese population and compare them with other populations.

## Methods

The present study was conducted from January 2010 to January 2012 at the Department of Forensic Medicine, Faculty of Medicine and Allied Sciences, Rajarata University of Sri Lanka.

Ethical clearance for this study was obtained from the Ethical Clearance Committee of the institute. All subjects were informed about the purpose, nature and possible risks of the study, before written informed consent was obtained.

The participants in this study were undergraduate students from different faculties in the university. We calculated that a sample size of 434 participants was sufficient to detect a 50% prevalence of ulnar loop, with an absolute precision of 5% of the total population (according to the 2009 census) [19]. There are nine provinces in Sri Lanka. The proportional quota for each province was calculated based on the population percentage in each province. Subsequently, the students were selected based on their inhabitant province. Nonresident Sinhalese, other nationalities (Sri Lankan Tamil, Sri Lankan Moor, Indian Tamil and other ethnic groups) and those with disease or deformity of the fingers were excluded from the study. Demographic details were obtained by interviewer-administered questionnaire. These details included age, gender and place of origin (province of residence). Eligible students were asked to wash their hands thoroughly to remove dirt, and to dry them before obtaining fingerprints. Rolled prints were obtained by the ink and paper method as described by Cummins and Midlo [2,20]. Digital prints of all ten fingers were obtained for each individual.

Digital prints were classified according to the Galton-Henry system [21,22]. We carefully examined digital prints to identify the following patterns, using a hand lens (magnification 10×)

1. Loops

● Ulnar loop (UL)

● Radial loop (RL)

2. Whorls

● Plain whorl (PW)

● Double loop whorl (DLW)

● Central pocket loop (CPL)

● Accidental whorl (AW)

3. Arches

● Plain arch (PA)

● Tented arch (TA)

The pattern intensity index:

{(2 ×% whorl +% loop) ÷ 2} [23,24];

arch/whorl index of Dankmeijer:

{(% arches ÷% whorl) × 100} [25];

and whorl/loop index of Furuhata:

{(% whorl ÷% of loop) × 100} [26], were calculated.

Analysis was carried out using SPSS 17(SPSS Inc. Released 2008. SPSS Statistics for Windows, Version 17.0. Chicago: SPSS Inc.) Categorical data are presented as frequencies.

## Results

A total of 4,340 fingerprints from 434 Sri Lankan Sinhalese (217 males and 217 females) were analyzed for different digital patterns. The mean age of the group was 23.66 years (standard deviation = ±4.93 years).

**Figure 1:**
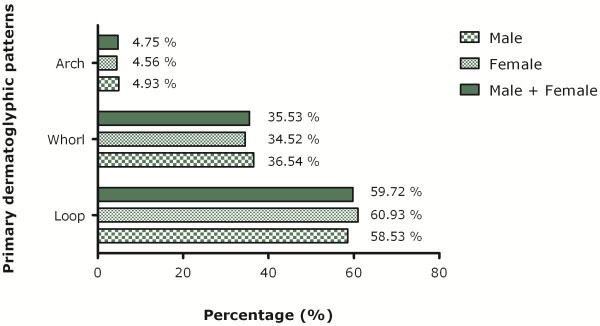
Digital dermatoglyphics patterns distribution among Sinhalese.

The loop pattern (n = 2,592, 59.72%) is the most common pattern in the Sinhalese population followed by whorl (n = 1,542, 35.53%) and arch (n = 206, 4.75%) (Figure 1).

Diversity of digital dermatoglyphic traits between males and females is shown in Tables 1 and 2 respectively.

**Table 1:** Frequency (%) of digital dermatoglyphic pattern in males

**Digit**	**Hand**	**Loop**		**Whorl**				**Arch**	
		**Ulnar loop**	**Radial loop**	**Plain whorl**	**Double loop whorl**	**Central pocket loop**	**Accidental whorl**	**Plain arch**	**Tented arch**
I (Thumb)	R	47.93	0.46	29.49	17.51	0.46	1.38	2.3	0.46
	L	53.46	0.92	23.04	17.51	0	2.76	1.38	0.92
	R + L	50.69	0.69	26.27	17.51	0.23	2.07	1.84	0.69
II (Index finger)	R	45.62	5.53	28.11	3.69	3.69	0.92	5.53	6.91
	L	38.25	7.83	32.72	2.3	3.23	1.38	6.91	7.37
	R + L	41.94	6.68	30.41	3	3.46	1.15	6.22	7.14
III (Middle finger)	R	70.97	0.92	16.13	5.07	0.92	1.38	3.69	0.92
	L	64.52	0.92	19.35	4.15	0.92	0.92	5.07	4.15
	R + L	67.74	0.92	17.74	4.61	0.92	1.15	4.38	2.53
IV (Ring finger)	R	38.25	0.92	47.93	0.92	9.68	0.92	0.92	0.46
	L	45.62	0	39.17	5.53	8.29	0	0.92	0.46
	R + L	41.94	0.46	43.55	3.23	8.99	0.46	0.92	0.46
V (Little finger)	R	82.49	0.46	11.98	0.92	4.15	0	0	0
	L	80.18	0	10.14	3.69	3.69	1.38	0.46	0.46
	R + L	81.34	0.23	11.06	2.3	3.92	0.69	0.23	0.23
All digits	R	57.05	1.66	26.73	5.62	3.78	0.92	2.49	1.75
	L	56.41	1.93	24.88	6.64	3.23	1.29	2.95	2.67
	R + L	56.73	1.80	25.81	6.13	3.50	1.10	2.72	2.21

R = Right, L = Left.

**Table 2:** Frequency (%) of digital dermatoglyphic pattern in females

**Digit**	**Hand**	**Loop**		**Whorl**				**Arch**	
		**Ulnar loop**	**Radial loop**	**Plain whorl**	**Double loop whorl**	**Central pocket loop**	**Accidental whorl**	**Plain arch**	**Tented arch**
I (Thumb)	R	53.46	0	25.35	16.13	0.46	1.84	2.3	0.46
	L	48.85	2.3	23.04	17.97	0.92	3.23	3.69	0
	R + L	51.15	1.15	24.19	17.05	0.69	2.53	3	0.23
II (Index finger)	R	51.61	0.92	24.42	8.76	3.69	2.3	3.69	4.61
	L	34.56	13.36	29.95	5.99	4.15	1.38	4.61	5.99
	R + L	43.09	7.14	27.19	7.37	3.92	1.84	4.15	5.3
III (Middle finger)	R	77.42	0.92	13.36	3.69	0.46	1.38	0.92	1.84
	L	59.91	2.76	20.74	3.23	3.23	1.38	4.15	4.61
	R + L	68.66	1.84	17.05	3.46	1.84	1.38	2.53	3.23
IV (Ring finger)	R	53.46	0	38.71	1.84	4.61	0	0.92	0.46
	L	48.85	0.92	37.79	1.84	5.99	2.3	0.92	1.38
	R + L	51.15	0.46	38.25	1.84	5.3	1.15	0.92	0.92
V (Little finger)	R	81.57	0.46	11.06	0.46	3.69	0	0.46	2.3
	L	77.42	0.46	12.44	0.92	6.45	0	0.46	1.84
	R + L	79.49	0.46	11.75	0.69	5.07	0	0.46	2.07
All digits	R	63.50	0.46	22.58	6.18	2.58	1.10	1.66	1.93
	L	53.92	3.96	24.79	5.99	4.15	1.66	2.77	2.76
	R + L	58.71	2.21	23.69	6.08	3.36	1.38	2.21	2.35

R = Right, L = Left.

The loop pattern is the most frequently observed pattern in both hands of females (right hand = 63.96%, left hand 57.88%), Similarly, the most frequently observed pattern in both hands of males (right hand 58.71%, left hand 58.34%) is also loop. However, the overall frequency of loop pattern is higher in females (60.92%) than males (58.53%). The whorl pattern is observed more frequently in males (36.54%) compared to females (34.52%). The frequency of arch pattern is 4.56% in females and 4.93% in males (Figure 1).

In detail, the most frequently observed pattern type is ulnar loop (females 58.71%, males 56.73%) for both hands, followed by plain whorl (females 23.69%, males 25.81%), double loop whorl (females 6.08%, males 6.13%), central pocket loop (females 3.36%, males 3.50%), plain arch (females 2.21%, males 2.72%), tented arch (females 2.35%, males 2.21%), radial loop (females 2.21%, males 1.80%) and accidental whorl (females 1.38%, males 1.10%).

In the left hands of all subjects,the pattern distribution in descending order is: ulnar loop (females 53.92%, males 56.41%), plain whorl (females 24.79%, males 24.88%), double loop whorl (females 5.99%, males 6.44%), central pocket loop (females 4.15%, males 3.23%), radial loop (females 3.96%, males 1.93%), plain arch (females 2.77%, males 2.95%), tented arch (females 2.76%, males 2.67%) and accidental whorl (females 1.66%, males 1.29%). Similarly, for the right hand, the distribution is: ulnar loop (females 63.50%, males 57.05%), plain whorl (females 22.58%, males 26.73%), double loop whorl (females 6.18%, males 5.62%), central pocket loop (females 2.58%, males 3.78%), plain arch (females 1.66%, males 2.49%), tented arch (females 1.93%, males 1.75%), radial loop (females 0.46%, males 1.66%), and accidental whorl (females 1.10%, males 0.92%).

The decreasing order of digital dermatoglyphic pattern types from finger to finger is shown in Table 3.

**Table 3:** Frequency (%) of digital dermatoglyphic patterns of fingers in descending order

**Loop pattern**		
Male	Right hand	V (82.95%) > III (71.89%) > II (51.15%) > I (48.39%) > II (39.17%)
	Left hand	V (80.18%) > III (64.52%) > I (53.46%) > IV (45.62%) > II (38.25%)
Female	Right hand	V (82.03%) > III (78.34%) > I (53.46%) and IV (53.46%) > II (52.53%)
	Left hand	V (77.88%) > III (62.67%) > I (51.15%) > IV (49.77%) > II (47.92%)
**Whorl pattern**		
Male	Right hand	IV (59.45%) > I (48.84%) > II (36.41%) > III (23.50%) > V (17.05%)
	Left hand	IV (52.99%) > I (43.31%) > II (39.63%) > III (25.34%) > V (18.90%)
Female	Right hand	IV (45.16%) > I (43.78%) > II (39.17%) > III (18.89%) > V (15.21%)
	Left hand	IV (47.92%) > I (45.16%) > II (41.47%) > III (28.58%) > V (19.81%)
**Arch pattern**		
Male	Right hand	II (12.44%) > III (4.61%) > I (2.76%) > IV (1.38%) > V (0.00%)
	Left hand	II (14.28%) > III (9.22%) > I (2.30%) > IV (1.38%) > V (0.92%)
Female	Right hand	II (8.30%) > III (2.76%) and I (2.76%) and V (2.76%) > IV (1.38%)
	Left hand	II (10.60%) > III (8.76%) > I (3.69%) > IV (2.30%) > V (2.30%)

I = Thumb, II = Index finger, III = Middle finger, IV = Ring finger, V = Little finger.

The double loop whorls are found more frequently on the thumb (males; right 17.51%, left 17.51% females; right 16.13%, left 17.97%) than on the other fingers ((index finger (males; right 3.69%, left 2.3%, females; right 8.76%, left 5.99%), middle finger (males; right 5.07%, left 4.15% females; right 3.69%, left 3.23%), ring finger (males; right 0.92%, left 5.53% females; right 1.84%, left 1.84%), little finger (males; right 0.92%, left 3.69% females; right 0.46%, left 0.92%)).

The frequencies of dermatoglyphic pattern indices among Sinhalese are shown in Table 4.

**Table 4:** Pattern indices of Sinhalese

**Population**	**Gender**	**Index of pattern intensity**^**a**^	**Index of Dankmeijer**^**b**^	**Index of Furuhata**^**c**^
Sinhalese (Sri Lanka)	Male	13.16	13.49	62.44
	Female	12.99	13.22	56.65
	M + F	13.08	13.35	59.55

^a^The pattern intensity index = *(2 ×% whorl +% loop) ÷ 2.*

^b^Dankmeijer index = *(% arches ÷% whorl) × 100.*

^c^Furuhata index = *(% whorl ÷% of loop) × 100.*

The pattern intensity index is found higher in males (13.16) compared to females (12.99). Similarly, the index of Dankmeijer is found higher in males (13.49) than females (13.22). The index of Furuhata is found higher in males (62.44) compared to females (56.65).

## Discussion

In this study, an attempt has been made to study the sexual dimorphism of dermatological traits and pattern indices among a sample of Sinhalese in Sri Lanka. They are typified by having a high frequency of loops compared to whorls and arches. Ulnar loop is the most commonly observed pattern followed by PW, DLW, CPL, PA, TA, RL and AW in males and similarly, UL is the commonest pattern followed by PW, DLW, CPL, TA, PA, RL and AW in females.

A large number of dermatoglyphics studies have been performed over the last century in many countries around the world. The results of the following studies are in line with the present study (Table 5).

**Table 5:** A comparison of the dermatoglyphic patterns of Sinhalese with several other populations

**Population**	**Sex**	**N**	**Frequency of dermatoglyphic patterns (%)**											**Authors**
			**Loop**			**Whorl**					**Arch**			
			**UL**	**RL**	**Total**	**PW**	**DLW**	**CPL**	**AW**	**Total**	**PA**	**TA**	**Total**	
**Sinhalese (Sri Lanka)**	M	217	56.73	1.79	58.52	25.8	6.13	3.5	1.1	36.54	2.72	2.21	4.93	Present study
	F	217	58.7	2.21	60.92	23.69	6.08	3.36	1.38	34.52	2.21	2.35	4.56	
**RarhiBrahmins (Bengal)**	M	100			53.8					43.9			2.3	Chattopadhyay *et al*. [6]
	F	38			64.47					31.32			4.21	
**Tunisians (Tunisia)**	M	233			61.72					31.31			7.08	Namouchi [16]
	F	110			63.54					27.74			8.63	
**DanguriaTharu of Uttar Pradesh (India)**	M	379	52.92	1.76	54.69	32.71	5.67	2.98	0.05	41.42	3	0.87	3.87	Srivastava [10]
	F	300	53.76	1.56	55.33	33.16	4.5	2.8	0.03	40.5	3.86	0.3	4.16	
**Black Americans (USA)**	M	100	61	2.1	63.1					33.6			3.3	Qazi *et al*. [17]
	F	100	58	1.7	59.7					31.3			8.2	
**South Indians (India)**	M	250	49.32	2.08	51.4	30.64	6.24	3.72	0.48	41.08	3.6	2.08	5.68	Nithin *et al*. [9]
	F	250	36.8	1.36	38.16	26.84	5.48	2.68	0.36	35.36	2.36	2.16	4.52	
**Nigerians (Nigeria)**	M	400	52.76	1.38	54.14					30.05			16	Boroffice [18]
	F	400	51.43	0.88	52.31					25.3			22.4	
**Indigenous black Zimbabweans (Zimbabwe)**	M	135	72.22^a^	5.55^a^	77.77^a^	-	-	-	-	12.23^a^	-	-	10^a^	Igbigbi [14]
	F	135	78.33^a^	6.67^a^	85^a^	-	-	-	-	5^a^	-	-	10^a^	
**RengmaNagas of Nagaland (India)**	M	104	43.96	3.36	46.96	-	-	-	-	52.19	-	-	0.49	Banik *et al*. [8]
	F	103	40.58	1.94	42.52	-	-	-	-	55.69	-	-	1.79	
**Dhimals of North Bengal(Bengal)**	M	101	41.37	0.78	42.16	32.55	5.88	16.67	0	55.10	1.96	0.78	2.75	Biswas [7]
	F	101	46.08	2.16	48.24	27.84	6.08	16.27	0	50.19	0	1.57	1.57	
**Tibetans (Tibet)**	M	156	36.83	2.16	38.99	48.98	8.35	2.84	0.06	60.24	0.51	0.26	0.76	Tiwari *et al*. [11]
	F	150	47.13	2	49.13	39.6	6.6	2.4	0.07	48.67	2.07	0.13	2.2	
**Muzziena Bedouin (South Sinai** ***)***	M	170	46.3	2.9	49.2					49.1			1.7	Karmakar *et al*. [15]
	F	48	45.8	2.4	48.2					50.3			1.6	
**Samoan New Zealanders (New Zealand** ***)***	M	100	42.8	0.8	43.6	35.3	18.1	1.9	0.3	55.6	0.6	0.2	0.8	Cho [12]
	F	93	33.2	0.5	33.7	49.4	14.6	1.6	0	65.6	0.5	0.2	0.7	
**Australian Aborigines in the Northern Territory (Australia)**	M	114			42.6					56.7				Cho [13]
	F	90			47					51.2				

AW = Accidental whorl, CPL = Central pocket loop, DLW = Double loop whorl, PA = Plain arch, PW = Plain whorl, RL = Radial loop, TA = Tented arch, UL = Ulnar loop. ^a^Mean percentage.

Srivastava [10], in his study on the DanguriaTharu of Uttar Pradesh, found that, the UL was the most frequently observed pattern followed by PW, DLW, PA, CPL, RL, TA and AW in decreasing order of frequency in males, whereas in females UL was the most common pattern followed by PW, DLW, PA, CPL, RL, TA and AW in decreasing order of frequency. Nithin *et al. *[9]*,* in their study on South Indian people, observed that UL was the most common pattern followed by PW, DLW, CPL, PA, TA, RL and AW in males while UL was the most common pattern followed by PW, DLW, CPL, PA, TA, RL and AW in females. Similarly, studies done by Chattopadhyay *et al.*(among Rarhi Brahmins of Bengal) [6], Namouchi (among Tunisians) [16], Qazi *et al. *(among Black Americans) [17], Boroffice (among Nigerians) [18] and Igbigbi *et al. *(among Indigenous black Zimbabweans) [14], observed that loop was the most common pattern followed by whorl and arch in both hands of males and females.

The results of the studies done by Banik *et al. *(among RengmaNagas of Nagaland) [8], Biswas (among Dhimals of North Bengal) [7], Tiwari *et al. *(among Tibetans) [11], Karmakar *et al. *(among Muzziena Bedouin) [15] and Cho (among Samoan New Zealanders and Australian Aborigines in the Northern Territory) [12,13] are not substantiated with the current study. They observed whorls as the most common pattern, followed by loops and arches in both hands of male and females. Differences in heritability and developmental variation among sexes might account for sexual dimorphism of these patterns [27]. According to the generalization of Cummins and Midlo, it is expected that whorl patterns and radial loops should occur more commonly on the right hand digits in both sexes compared to the left hand digits [2]. However, the average radial loop percentage found in left hands of both Sinhalese males and females is higher compared to right hands. The whorl percentage is higher in left hands than right hands in females. Whorl percentage was higher in right hands compared to left hands in males. Double loop whorl pattern is observed more frequently in digit 1 of both hands compared to other fingers among Sinhalese. Holt [28] (as cited in Karmakar *et al*. [29]) stated that ‘certain patterns tend to occur more frequently on some digit than on others, which seems to be constant for any population’.

The pattern indices of Sinhalese are compared with several previous studies on different populations in Table 6. Pattern intensity index, Dankmeijer index and Furuhata index are higher in Sinhalese males compared to Sinhalese females. Studies by Cho [12] and Banik *et al.*[8] observed higher pattern intensity index in females. Tiwari *et al.*[11] and Biswas [7]observed higher pattern intensity index in males.

**Table 6:** A comparison of the dermatoglyphic pattern indices of Sinhalese with several other populations

**Population**	**Gender**	**Index of pattern intensity**	**Index of Dankmeijer**	**Index of Furuhata**	**Author**
Sinhalese (Sri Lanka)	Male	13.16	13.49	62.44	Present study
	Female	12.99	13.22	56.65	
	M + F	13.08	13.35	59.55	
Samoan New Zealanders (New Zealand)	Male	15.18	1.44	127.52	Cho [12]
	Female	16.49	1.07	194.66	
	M + F	15.99	1.32	156.59	
Tibetans (Tibet)	Male	15.95	1.26	154.5	Tiwari *et al*. [11]
	Female	14.65	4.5	99.06	
	M + F	15.3	2.88	126.78	
RengmaNagas of Nagaland (India)	Male	1.54	0.14	-	Banik *et al*. [8]
	Female	1.56	3.34	-	
	M + F	0.47	1.47	-	
Dhimals of North Bengal (Bengal)	Male	15.24	4.98	130.7	Biswas [7]
	Female	14.86	3.13	104.07	
	M + F	15.05	4.1	116.49	

Studies done by Biswas [7] and Cho [12] observed higher Dankmeijer index in males whereas Banik *et al.*[8] and Tiwari *et al.*[11] observed higher Dankmeijer index in females. A higher Furuhatas index was observed among males in the studies done by Tiwari *et al.*[11] and Biswas [7], whereas Cho [12] found a higher Furuhatas index among females.

In general, dermatoglyphics patterns of Sinhalese are more similar to the Caucasoid populations. The origin of the Sinhalese population of Sri Lanka is disputed. However, studies based on human leukocyte antigen (HLA) have shown that Sinhalese are more likely to originate from the Aryans than the Dravidians [30]. Sinhalese are genetically closer to Caucasoid populations than to other neighboring Mongoloid populations [31]. The history of Sri Lanka has been based on the *Mahavansa*, the great chronicle of Sri Lanka, which was written by the Mahanama thero in the fifth century AD [32]. According to the *Mahavansa*, Sinhalese people originated from a group of 700 people of Indo-Aryan stock led by Prince Vijaya (543 BC to 505 BC), who was a son of the North Indian king, Sinhabahu [33].

It appears that the dermatoglyphic data would certainly support similarities between the Sinhalese and people of North India.

## Conclusion

In conclusion, the most common fingerprint pattern observed among Sinhalese is ulnar loop. All pattern indices are found to be higher in males. To some extent, the dermatoglyphic patterns of the Sinhalese are similar to North Indians and other Caucasoid populations. Further studies with larger sample sizes are needed to substantiate our findings.

## Abbreviations

AW: Accidental whorl; CPL: Central pocket loop; DLW: Double loop whorl; PA: Plain arch; PW: Plain whorl; RL: Radial loop; TA: Tented arch; UL: Ulnar loop; HLA: Human leukocyte antigen; AD: Anno Domini; BC: Before Christ.

## Competing interests

The authors declare that they have no competing interests.

## Authors’ contributions

BTBW and GKR carried out the design of the study and performed the statistical analysis, interpretation of data, and drafting of the manuscript. All authors participated in collecting data, editing the manuscript and helped coordinate research activities. All authors read and approved the final manuscript.

## References

[B1] Mange AP Mange EJ Genetics: Human Aspects 1990 2 Sunderland, Massachusetts: Sinauer Associates

[B2] Cummins H Midlo C Finger Prints, Palms and Soles: An Introduction to Dermatoglyphics 1961 New York: Dover Publications

[B3] Galton F Finger Prints 1892 London: Macmillan and Company

[B4] Miličić J Pavićević R Halbauer MSB Durham NM, Fox KH, Plato CC Analysis of qualitative dermatoglyphic traits of the digito-palmar complex in carcinomas The state of dermatoglyphics: the science of finger and palm prints 2000 New York: Edwin Mellen Press 384

[B5] Schaumann BA Opitz JM Clinical aspects of dermatoglyphics Birth Defects Orig Artic Ser 1991 32 193 228 1786352

[B6] Chattopadhyay PK Sharma PD Finger dermatoglyphics of the Rarhi Brahmins of Bengal Am J Phys Anthropol 1969 32 397 401 10.1002/ajpa.1330300309 5791016

[B7] Biswas S Finger and palmar dermatoglyphicstudy among the Dhimals of North Bengal, India Anthropologist 2011 32 235 238

[B8] Banik SD Pal P Mukherjee DP Finger dermatoglyphicvariations in RengmaNagas of Nagaland India Coll Antropol 2009 32 31 35 19408600

[B9] Nithin MD Balaraj BM Manjunatha B Mestri SC Study of fingerprint classification and their gender distribution among South Indian population J Forensic Leg Med 2009 32 460 463 10.1016/j.jflm.2009.07.001 19782316

[B10] Srivastava RP A study of finger prints of the DanguriaTharu of Uttar Pradesh (India) Am J Phys Anthropol 1963 32 69 76 10.1002/ajpa.1330210109 13978611

[B11] Tiwari SC Chattopadhyay PK Finger dermatoglyphics of the Tibetans Am J Phys Anthropol 1967 32 289 296 10.1002/ajpa.1330260303 6035854

[B12] Cho C A finger dermatoglyphics of the New Zealand-Samoans Korean J Biol Sci 1998 32 507 511 10.1080/12265071.1998.9647453

[B13] Cho C Finger dermatoglyphics of Australian Aborigines in the Northern Territory of Australia Korean J Biol Sci 2000 32 91 94 10.1080/12265071.2000.9647529

[B14] Igbigbi PS Msamati BC others Palmar and digital dermatoglyphics of Indigenous black Zimbabweans Med Sci Monit Int Med J Exp Clin Res 2002 32 CR757 12444380

[B15] Karmakar B Kobyliansky E Finger and palmar dermatoglyphics in Muzzeina Bedouin from South Sinai: a quantitative study Pap Anthropol 2012 32 110 122

[B16] Namouchi I Anthropological significance of dermatoglyphic trait variation: an intra-Tunisian population analysis Int J Mod Anthropol 2011 32 12 27

[B17] Qazi QH Mapa HC Woods J Dermatoglyphics of American blacks Am J Phys Anthropol 1977 32 483 487 10.1002/ajpa.1330470321 931027

[B18] Boroffice RA Digital dermatoglyphic patterns in a sample of the Nigerian population Am J Phys Anthropol 1978 32 167 169 10.1002/ajpa.1330490203 717551

[B19] Department of census and statistics, Sri Lanka http://www.statistics.gov.lk

[B20] Recording legible fingerprints http://www.fbi.gov/about-us/cjis/fingerprints_biometrics/recording-legible-fingerprints/takingfps

[B21] Cole SA Suspect Identities: A History of Fingerprinting and Criminal Identification 2002 Cambridge, Massachusetts: Harvard University Press

[B22] United States The Science of Fingerprints Classification and Uses 2006 Washington, DC: United States Department of Justice 209

[B23] Cummins H Steggerda M Finger prints in a Dutch family series Am J Phys Anthropol 1935 32 19 41 10.1002/ajpa.1330200106

[B24] Basu A Namboodiri KK The relationship between total ridge count and pattern intensity index of digital dermatoglyphics Am J Phys Anthropol 1971 32 165 173 10.1002/ajpa.1330340203 5572601

[B25] Dankmeijer J Some anthropological data on finger prints Am J Phys Anthropol 1938 32 377 388 10.1002/ajpa.1330230402

[B26] Furuhata T The difference of the index of finger prints according to race Japan Med World 1927 32 162 164

[B27] Meier RJ Anthropological dermatoglyphics: a review Am J Phys Anthropol 1980 32 147 178 10.1002/ajpa.1330230509

[B28] Holt SB The Genetics of Dermal Ridges 1968 Charles C Thomas: Springfield

[B29] Karmakar B Yakovenko K Kobyliansky E Qualitative finger and palmar dermatoglyphics: sexual dimorphism in the Chuvashian population of Russia Anthropol Anzeiger 2007 32 383 390 18196762

[B30] Malavige GN Rostron T Seneviratne SL Fernando S Sivayogan S Wijewickrama A Ogg GS HLA analysis of Sri Lankan Sinhalese predicts North Indian origin Int J Immunogenet 2007 32 313 315 10.1111/j.1744-313X.2007.00698.x 17845299

[B31] Roychoudhury AK Nei M Genetic relationships between Indians and their neighboring populations Hum Hered 1985 32 201 206 10.1159/000153545 4029959

[B32] Mahavamsa https://en.wikipedia.org/wiki/Mahavamsa

[B33] Prince Vijaya https://en.wikipedia.org/wiki/King_Vijaya

